# Machine learning models to predict outcomes at 30‐days using Global Leadership Initiative on Malnutrition combinations with and without muscle mass in people with cancer

**DOI:** 10.1002/jcsm.13259

**Published:** 2023-05-31

**Authors:** Nicole Kiss, Belinda Steer, Marian de van der Schueren, Jenelle Loeliger, Roohallah Alizadehsani, Lara Edbrooke, Irene Deftereos, Erin Laing, Abbas Khosravi

**Affiliations:** ^1^ Institute for Physical Activity and Nutrition Deakin University Geelong Australia; ^2^ Department of Allied Health Peter MacCallum Cancer Centre Melbourne Australia; ^3^ Department of Nutrition and Speech Pathology Peter MacCallum Cancer Centre Melbourne Australia; ^4^ Department of Nutrition, Dietetics and Lifestyle HAN University of Applied Sciences Nijmegen The Netherlands; ^5^ Department of Human Nutrition and Health Wageningen University and Research Wageningen The Netherlands; ^6^ Institute for Intelligent Systems Research and Innovation Deakin University Waurn Ponds Australia; ^7^ Department of Health Services Research Peter MacCallum Cancer Centre Melbourne Australia; ^8^ Department of Physiotherapy The University of Melbourne Parkville Australia; ^9^ Department of Surgery, Western Health The University of Melbourne Parkville Australia; ^10^ Department of Nutrition and Dietetics Western Health, Footscray Australia

**Keywords:** Cancer, GLIM, Malnutrition, Muscle mass, Validation

## Abstract

**Background:**

Equipment to assess muscle mass is not available in all health services. Yet we have limited understanding of whether applying the Global Leadership Initiative on Malnutrition (GLIM) criteria without an assessment of muscle mass affects the ability to predict adverse outcomes. This study used machine learning to determine which combinations of GLIM phenotypic and etiologic criteria are most important for the prediction of 30‐day mortality and unplanned admission using combinations including and excluding low muscle mass.

**Methods:**

In a cohort of 2801 participants from two cancer malnutrition point prevalence studies, we applied the GLIM criteria with and without muscle mass. Phenotypic criteria were assessed using ≥5% unintentional weight loss, body mass index, subjective assessment of muscle stores from the PG‐SGA. Aetiologic criteria included self‐reported reduced food intake and inflammation (metastatic disease). Machine learning approaches were applied to predict 30‐day mortality and unplanned admission using models with and without muscle mass.

**Results:**

Participants with missing data were excluded, leaving 2494 for analysis [49.6% male, mean (SD) age: 62.3 (14.2) years]. Malnutrition prevalence was 19.5% and 17.5% when muscle mass was included and excluded, respectively. However, 48 (10%) of malnourished participants were missed if muscle mass was excluded. For the nine GLIM combinations that excluded low muscle mass the most important combinations to predict mortality were (1) weight loss and inflammation and (2) weight loss and reduced food intake. Machine learning metrics were similar in models excluding or including muscle mass to predict mortality (average accuracy: 84% vs. 88%; average sensitivity: 41% vs. 38%; average specificity: 85% vs. 89%). Weight loss and reduced food intake was the most important combination to predict unplanned hospital admission. Machine learning metrics were almost identical in models excluding or including muscle mass to predict unplanned hospital admission, with small differences observed only if reported to one decimal place (average accuracy: 77% vs. 77%; average sensitivity: 29% vs. 29%; average specificity: 84% vs. 84%).

**Conclusions:**

Our results indicate predictive ability is maintained, although the ability to identify all malnourished patients is compromised, when muscle mass is excluded from the GLIM diagnosis. This has important implications for assessment in health services where equipment to assess muscle mass is not available. Our findings support the robustness of the GLIM approach and an ability to apply some flexibility in excluding certain phenotypic or aetiologic components if necessary, although some cases will be missed.

## Introduction

The Global Leadership Initiative on Malnutrition (GLIM) criteria are the result of consensus between global nutrition societies on the core diagnostic criteria for malnutrition in clinical settings.^1^ Three phenotypic criteria, low muscle mass, low body mass index (BMI) or unintentional loss of weight, and two aetiologic criteria, reduced food intake or inflammation, were chosen based on their relevance to malnutrition and ability individually to predict adverse outcomes.^1^

Since publication of the GLIM criteria in 2018, numerous studies in people with cancer have investigated the ability of the GLIM criteria to predict adverse clinical outcomes using a variety of techniques to determine low muscle mass. In overweight people following surgery for gastric cancer, Huang et al. reported GLIM defined malnutrition, using computed tomography images to determine loss of muscle mass, was not predictive of overall and disease‐free survival unless used in combination with gait speed and handgrip strength.^2^ Anthropometric techniques, such as calf circumference, have also been used to assess low muscle mass within the GLIM criteria and are predictive of shorter survival in people with lung cancer.^3^ While in cancer inpatients, a diagnosis of malnutrition using GLIM with reduced muscle mass determined from fat‐free mass index, was associated with increased mortality at 6 months.^4^ Similar studies of different muscle mass assessments in applying the GLIM criteria were also associated with higher mortality in older adults without cancer.^5^, ^6^

Establishing that a range of techniques can be used to assess muscle mass in application of the GLIM criteria is important to provide flexibility in clinical practice. However, tools to assess muscle mass may not be available or routine practice in all health services due to resource limitations or a lack of knowledge and training. Currently, we have no understanding whether applying the GLIM criteria without an assessment of muscle mass, and using only body mass index or unintentional weight loss as the phenotypic criteria, affects the ability to predict adverse outcomes. This study used machine learning approaches to determine which combinations of GLIM phenotypic and aetiologic criteria are most important for the prediction of 30‐day mortality and unplanned admission using combinations including and excluding low muscle mass.

## Methods

### Study design

This study was a secondary analysis of data collected as part of two point prevalence studies (PPS) conducted from 14 November to 9 December 2016 and 9 July to 3 August 2018. There were 16 health services involved in 2016 and 19 acute hospitals involved in 2018, with the methods previously reported.^7^ Approval for the original PPS was obtained from the Human Research Ethics Committee at Peter MacCallum Cancer Centre, Melbourne, Australia (HREC/16/PMCC/149) and approval for the current analysis received exception from ethics review from Deakin University (2020‐289). All participants provided informed consent to participate in the PPS.

### Eligibility criteria

Eligible participants were ≥18 years; with a confirmed cancer diagnosis; admitted to hospital for cancer treatment or related management for a multiday stay; or ambulatory patients attending for radiotherapy, intravenous chemotherapy or immunotherapy. Patients who were not able to provide verbal consent; terminally ill with a life expectancy of <1 month; attending for medical review, day surgery, oral chemotherapy, maintenance or hormonal treatment only; admitted to intensive care or the emergency department on the day of data collection were excluded.

### Measures

#### Demographic and clinical data

Demographics data encompassing age, sex and living situation were recorded. Details of cancer diagnosis, treatment setting and location (metropolitan or rural), current cancer treatment (chemotherapy, surgery, radiotherapy, stem cell transplant, immunotherapy, or other cancer‐related management), and whether metastatic disease was present were collected.

#### Nutrition assessment and Global Leadership Initiative on Malnutrition parameters

Usual hospital equipment was used to obtain height and weight, or were patient reported, and subsequently used to calculate Body Mass Index (BMI, kg/m^2^). All participants were asked if they had experienced reduced food intake or weight loss. If weight loss was reported, participants were asked the timeframe over which it occurred (≤3 months or ≥4 months) and if the weight loss was intentional or unintentional. Participants reporting reduced food intake were asked the duration of the reduced intake (0–4 days, 5–30 days, > 1 month) and how much this had decreased compared with usual intake >75%, ≤75%, ≤50%, ≤25%). Screening for nutritional risk was completed using the validated Malnutrition Screening Tool (MST),^8^, ^9^ with a score of ≥2 on the MST indicating risk of malnutrition. For participants who were not at risk malnutrition (scored <2), no further nutrition assessment was completed as they were considered to be well‐nourished.^10^ A subjective physical examination of muscle mass at a minimum of four of seven sites used for the Patient‐Generated Subjective Global Assessment (PG‐SGA) was completed by a trained dietitian for participants who were at nutritional risk (scored ≥2).^11^, ^12^ Muscle sites assessed included the temples, clavicles, shoulders, interosseous, scapular, thigh and calf and each muscle site was graded as no muscle loss, mild/moderate or severe loss of muscle mass.^12^

**Figure 1:**
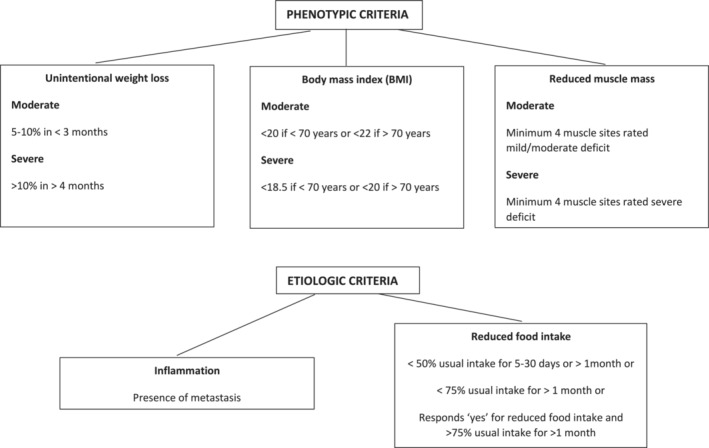
Data used to determine the GLIM criteria within this study.

Guidance for validation of the operational criteria for GLIM recommend not classifying all people with cancer as automatically having inflammation and instead suggest the use severity rating.^13^ An objective measure of inflammation was not available in this dataset and instead disease severity was rated according to the presence or absence of metastatic disease. Our group recently demonstrated that metastatic disease can be used as a proxy for inflammation in the absence of an objective measure.^14^ The data used to apply the GLIM phenotypic or aetiologic criteria and if malnutrition was moderate or severe are presented in Figure 1. There are 21 possible GLIM combinations, of at least one phenotypic and one aetiologic criteria, which we labelled GLIM 1 through to GLIM 21 and these are described in Data S1.

#### 30‐day outcomes

Mortality at 30 days and unplanned inpatient admission within the same period to the original hospital were collected from the medical record.

### Statistical analysis

SPSS version 26 (SPSS Inc, Chicago, IL, USA) was used for data analysis. Continuous variables were reported as means (standard deviation), and categorical variables were summarised as counts and percentages.

### Machine learning analysis

The methods used for the machine learning analysis have been reported in detail in a previous paper, including the approach using all 21 GLIM combinations.^14^ In brief, we used machine learning to determine the combinations of nine GLIM phenotypic and aetiologic criteria, excluding muscle mass, that were most predictive of mortality and unplanned admission or readmission. The machine learning models were generated by splitting the data into 75% for training and 25% for testing. The small proportion (2%) of participants who were deceased at 30 days resulted in a highly imbalanced dataset. The average performance metrics were determined through repeating the model training process 200 times. Accuracy represents the proportion of test samples that have been predicted correctly by the model and is calculated as (true positives + true negatives)/(true positives + true negatives + false positives + false negatives).^15^ The most important purpose of machine learning algorithms is to achieve high accuracy. Sensitivity and specificity metrics represent the percentage of positive and negative samples that have been predicted correctly, respectively. Sensitivity is calculated as true positives/(true positives + false negatives). Specificity is calculated as true negatives/(true negatives + false positives). The threshold for calculating the performance metrics (accuracy, sensitivity, and specificity) was set to 0.5.

We used three approaches. Firstly, the most important GLIM combinations were determined using decision trees. Secondly these decision trees were used to determine the probability of 30‐mortality or unplanned admission according to the present conditions. Finally, data were classified using random forest.^16^ Analyses were conducted using open‐sources Python packages including Pandas (data wrangling) and scikit‐learn (ML).

## Results

In total, 2801 people participated in the point prevalence studies, of which seven participants did not have data on risk of malnutrition and 302 had missing data for one or more of the GLIM phenotypic or aetiologic variables. This left 2492 participants to be included in the study analysis. Almost half (49%, *n* = 1229) of participants were 65 years or older, there was an even representation of males and females, and the vast majority were living with family, a carer or in residential care (Table 1). The most common cancer types were breast (19%), haematological (19%), and colorectal (14%) malignancies. Within the 30 days following initial data collection, 300 (12%) of participants had experienced an unplanned hospital admission, while 53 (2%) of participants were deceased.

**Table 1:** Participant characteristics (*N* = 2492)

Characteristic	*n* (%)
Age, years	
Mean (SD)	62.3 (14.2)
Sex	
Male	1237 (49.6)
Female	1254 (50.3)
Unknown	1 (0.1)
Living situation	
Living alone	449 (18.0)
Living with family/ carer/ residential care	2038 (81.8)
Unknown	5 (0.2)
Cancer diagnosis	
Bone and soft tissue	44 (2.0)
Breast	466 (19.0)
Central nervous system	35 (1.0)
Colorectal	355 (14.0)
Endocrine or thyroid	38 (1.5)
Genitourinary	197 (8.0)
Gynaecological	124 (5.0)
Haematological	466 (19.0)
Head and neck	163 (6.0)
Lung	273 (11.0)
Skin or melanoma	123 (5.0)
Upper gastrointestinal	171 (7.0)
Unknown primary	14 (0.6)
Other thoracic or abdominal	17 (0.7)
Unknown	6 (0.2)
Treatment type^a^	
Chemotherapy	1728 (69.0)
Radiotherapy	785 (31.0)
Surgery	582 (23.0)
Stem cell transplant	53 (2.0)
Immunotherapy	163 (6.0)
Other	193 (8.0)
Cancer‐related management	60 (2.0)
Type of admission	
Inpatient	513 (20.5)
Ambulatory patient	1979 (79.0)
Hospital location	
Metropolitan	2097 (84.1)
Rural	395 (15.9)

^a^ Multiple treatments selected for some participants. Values are *n* (%) unless specified.

### Malnutrition prevalence with and without muscle mass

The overall prevalence of malnutrition according to the GLIM criteria was 19.5% (*n* = 485/2492) as reported in a prior publication on this cohort.^14^ The prevalence of malnutrition using only GLIM phenotypic and aetiologic combinations that exclude muscle mass was 17.5% (437/2492), meaning 48 malnourished patients were not identified when muscle mass was excluded. The prevalence of the individual GLIM phenotypic and aetiologic variables are presented in Table 2.

**Table 2:** Prevalence of the individual phenotypic and aetiologic criteria (*N* = 2492)

Phenotypic criteria	*n* (%)
Reduced muscle mass	
Yes	378 (15)
No	2144 (85)
Low BMI	
Yes	147 (6)
No	2345 (94)
Unintentional weight loss	
Yes	499 (20)
No	1993 (80)

Aetiologic criteria	n (%)
Reduced food intake	
Yes	505 (20)
No	1987 (80)
Inflammation	
Yes	315 (13)
No	2177 (87)

Abbreviation: BMI, body mass index.

### Prediction of 30‐day mortality

**Figure 2:**
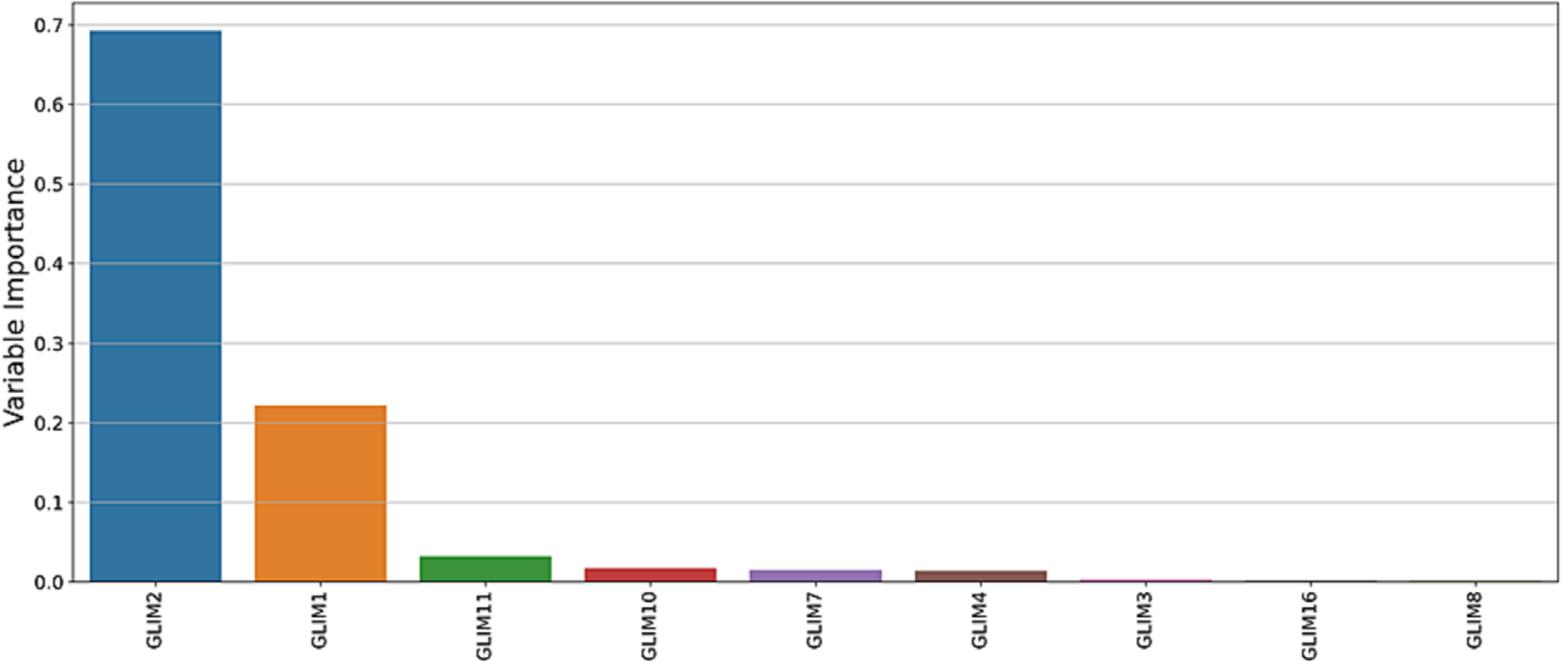
GLIM phenotypic and aetiologic combinations presented in order of importance to predict mortality within 30 days using the subset of nine GLIM combinations excluding the phenotypic criterion muscle mass.

Using the subset of nine GLIM diagnostic combinations (GLIM 1, 2, 3, 4, 7, 8, 10, 11, 16) to develop an entropy‐based decision tree, and based on the results of 200 training runs, GLIM 2 (weight loss and inflammation) and GLIM 1 (weight loss and reduced food intake) were the most important combinations to predict mortality within 30 days of data collection. Figure 2 shows, in order of importance, the GLIM combinations that were most important to predict 30‐day mortality. The machine learning model performed well with an average accuracy of 84%, average sensitivity of 41%, and average specificity of 85% to predict mortality within 30 days. This can be interpreted as follows: 84 out of 100 samples were correctly classified (accuracy), 40% of participants who died were correctly detected (sensitivity) and 85% of participants who did not die were correctly detected. Our previous publication reported the most important GLIM combinations and metrics of the machine learning model for all 21 GLIM combinations.^14^ Table 3 provides a comparison between the subset of nine GLIM combinations excluding muscle mass and all 21 GLIM combinations, demonstrating the performance of the model is largely unchanged using the subset excluding muscle mass.

**Table 3:** Comparison of most important GLIM combinations and machine learning model metrics using all 21 GLIM combinations and a subset excluding muscle mass to predict 30‐day mortality

Machine learning outcomes	Using a subset of GLIM combinations excluding muscle mass	Using all 21 GLIM combinations
Most important combinations	*GLIM 2* (weight loss, inflammation) *GLIM 1* (weight loss, reduced food intake)	*GLIM 12* (weight loss, reduced muscle mass, inflammation) *GLIM 13* (weight loss, reduced muscle mass, reduced food intake)
Average accuracy	84%	88%
Average sensitivity	41%	38%
Average specificity	85%	89%

Abbreviation: GLIM, Global Leadership Initiative on Malnutrition.

### Prediction of unplanned hospital admission within 30 days

**Figure 3:**
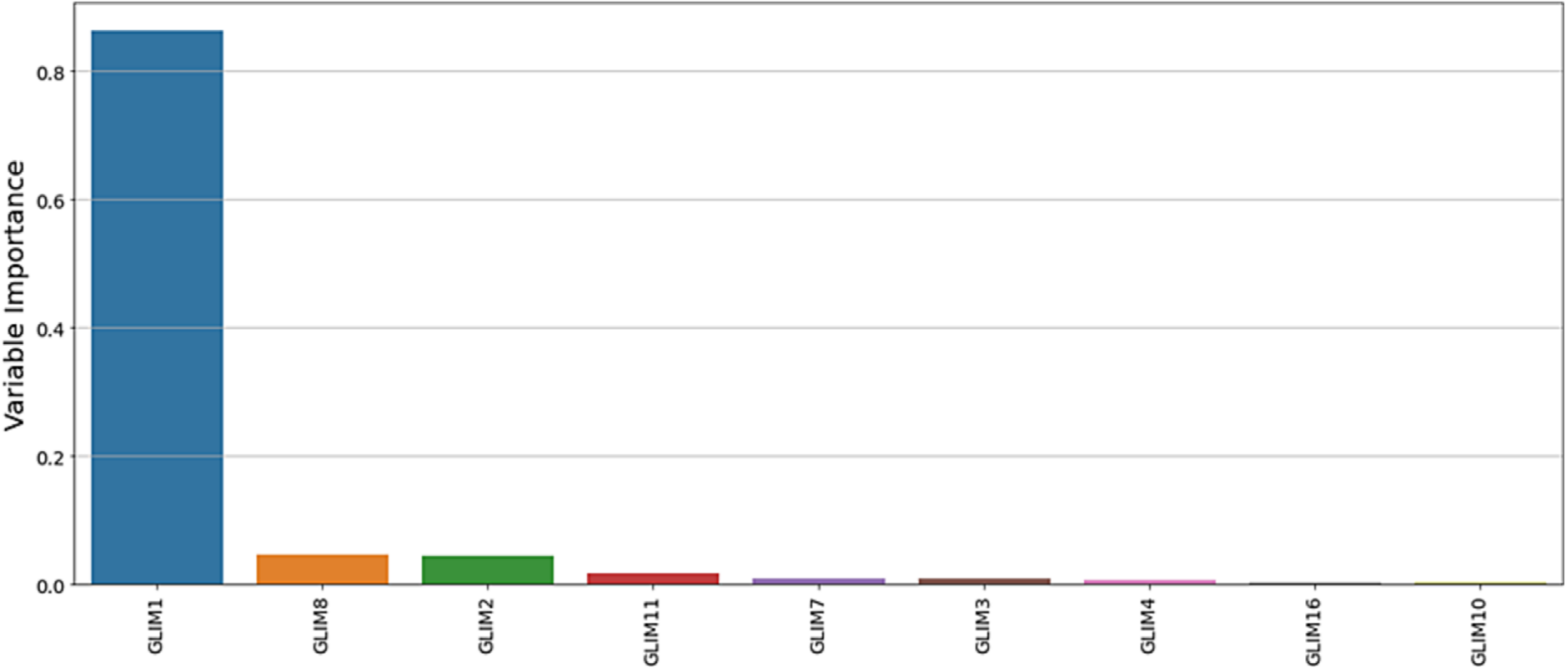
GLIM phenotypic and aetiologic combinations presented in order of importance to predict unplanned hospital admission within 30 days using the subset of nine GLIM combinations excluding the phenotypic criterion muscle mass.

Using the subset of nine GLIM combinations in the machine learning model and again based on the results of 200 training runs, GLIM 1 (weight loss and reduced food intake) was the most important combination to predict an unplanned hospital admission within 30 days of data collection. Figure 3 ranks, in order of importance, the GLIM combinations that predicted unplanned hospital admission within 30 days. The machine learning model performed well with an average accuracy of 77%, average sensitivity of 29%, and average specificity of 84% to predict unplanned admission. This can be interpreted as follows: 77 out of 100 samples were correctly classified (accuracy), 29% of participants who died were correctly detected (sensitivity) and 84% of participants who did not die were correctly detected. As reported above, our previous publication reported the most important GLIM combinations and metrics of the machine learning model for all 21 GLIM combinations.^14^ Table 4 compares the subset of nine GLIM combinations excluding muscle mass and all 21 GLIM combinations, demonstrating the performance of the model is unchanged using the subset excluding muscle mass.

**Table 4:** Comparison of most important GLIM combinations and machine learning model metrics using all 21 GLIM combinations or a subset excluding muscle mass to predict 30‐day unplanned hospital admission

Machine learning outcomes	Using a subset of GLIM combinations excluding muscle mass	Using all 21 GLIM combinations
Most important combinations	*GLIM 1* (weight loss, reduced food intake)	*GLIM 1* (weight loss, reduced food intake) *GLIM 5* (reduced muscle mass, reduced food intake)
Average accuracy^a^	77%	77%
Average sensitivity^a^	29%	29%
Average specificity^a^	84%	84%

Abbreviation: GLIM, Global Leadership Initiative on Malnutrition.

^a^ There were small differences in the average accuracy, sensitivity and specificity when using GLIM combinations excluding muscle mass compared with using all 21 combinations (e.g. accuracy 76.86% vs. 76.97%); however, these were rounded up for reporting.

## Discussion

In this study, 17.5% of participants were malnourished when assessed using only GLIM combinations that excluded muscle mass, in contrast to 19.5% when all GLIM combinations were used. This is lower than the 30 to 39% reported in previous point prevalence studies, and most likely reflects the low proportion of participants with metastatic disease (13%) in comparison to over 40% in these previous studies.^17^, ^18^ Unintentional weight loss was the most common of the phenotypic criteria, affecting one in five participants. We found unintentional weight loss with either inflammation or reduced food intake to be the most important combinations to predict 30‐day mortality when using only a subset of GLIM combinations that excluded muscle mass. However, unintentional weight loss plus reduced food intake was the only combination of importance to predict unplanned hospital admission within 30 days using the same subset of GLIM combinations.

In our cohort, weight loss and reduced muscle mass with either aetiologic criterion were the most important combinations to predict 30‐day mortality when all 21 GLIM combinations were utilised. When the machine learning model was applied to GLIM combinations that excluded reduced muscle mass, weight loss with either aetiologic criterion retained its importance in predicting 30‐day mortality. Notably, the machine learning metrics demonstrated minimal changes in the accuracy, sensitivity or specificity when reduced muscle mass was excluded. This indicates that if muscle mass is excluded from the GLIM assessment there is minimal impact on the ability to predict 30‐day mortality. In a prior publication we similarly found that machine learning models using GLIM combinations with and without inflammation performed almost identically in predicting 30‐day mortality.^14^ However, it must not be overlooked that excluding the assessment of muscle mass resulted in a substantial number of participants not being diagnosed with malnutrition. Meaning, while predictive ability is maintained when excluding an assessment of muscle mass, accuracy in identifying all cases of malnutrition appears to be compromised. This has significant implications for the assessment of malnutrition in clinical practice, particularly in health care environments where the equipment or skills to assess muscle mass are not available, resulting in some patients requiring intervention being missed. This suggests the weight loss phenotypic criterion has very similar predictive capability to the low muscle mass criterion, which further supports the robustness of the GLIM approach in considering multiple phenotypic criteria which are known to be independently associated with adverse outcomes related to malnutrition.^1^

Reduced muscle mass is clearly an important component of any assessment of malnutrition with the independent impact of reduced muscle mass on clinical outcomes, such as mortality, hospital admission, and post‐operative complications, well established.^19^, ^20^, ^21^ However, there is emerging evidence recently that suggests malnutrition, or other components of malnutrition such as weight loss, may confer a higher risk of mortality. In a retrospective study of 277 patients with head and neck cancer treated with (chemo)radiation malnutrition was independently associated with a 2.57 (95% CI 1.45, 4.55, *P* = 0.001) times greater risk of mortality, while computed tomography (CT) defined reduced muscle mass was not associated with mortality (HR 1.09, 95% CI 0.7, 1.71, *P* = 0.7).^22^ In an observational study of 1219 lung cancer patients, the GLIM criteria were applied and machine learning models were used to determine the individual phenotypic and aetiologic criteria most important for diagnosing and grading malnutrition.^23^ The authors report weight loss within 6 months was the most important variable for both diagnosis and grade of malnutrition. However, this emerging research suggests that assessing malnutrition, which is more comprehensive than assessment of reduced muscle mass alone, may be most important for identifying those at risk of adverse outcomes. This further emphasises that the exclusion of muscle mass measurement within a GLIM diagnosis may not affect the ability to predict people at higher risk of mortality. However, assessment of muscle mass, if equipment or skills are available, can support appropriately targeted nutritional therapy to treat muscle loss if present.

In contrast to 30‐day mortality, reduced food intake was the only aetiologic criterion that was important when predicting an unplanned hospital admission within 30 days. This was regardless of whether the full 21 GLIM combinations were used or the subset of nine combinations. There was no difference in the accuracy, sensitivity or specificity between the machine learning models using all versus the subset of GLIM combinations. Similar to mortality, this indicates that muscle mass may be excluded from the GLIM assessment without compromising the ability to determine patients at high risk of an unplanned admission.

It is important to note that we were unable to determine if the most important GLIM combinations to predict 30‐day mortality and unplanned admission were associated with a higher or lower probability of these adverse outcomes. This was the case also in our previous paper,^14^ and is due to high entropy in the nodes of the decision tree which classify participants into the predicted class (unplanned admission, no admission). Entropy is a value of one to zero and represents the degree of impurity in the decision tree node. When the entropy value is close to zero the majority of the samples in the node belong in the predicted class. However, if the entropy value is nearer to one this represents an even split where there is no ability to confidently predict the outcome. This may be improved through the inclusion of other variables such as treatment type, age, sex and disease stage in the machine learning model. However, our initial goal was to determine the relative importance of the various GLIM combinations to each other for predicting adverse outcomes, with and without muscle mass included. Likewise, the machine learning models had low sensitivity, ranging from 29% to 41%. This again may be improved by the inclusion of other variables in the model in future. Importantly, the accuracy of the models was high indicating the vast majority of samples were correctly classified. We have previously discussed that if accuracy of the machine learning models is high, the higher number of false positives produced by low sensitivity may not be as important.^14^ Generally, in healthcare, greater importance is placed on not missing patients at risk than on falsely identifying a patient at risk.

There are several strengths of this study which include the large and varied cancer cohort that support wide application of the results across a variety of healthcare settings and cancer diagnoses. Limitations of the study include the retrospective study design and secondary analysis of the data which meant that some variables, such as cancer stage, and longer‐term outcomes that may be important to consider were not available. There were some missing data and subsequently participants who were excluded from the analysis which may have affected the results. Some of the GLIM criterion were not applied exactly as per recommended, specifically metastatic disease is used as a proxy for inflammation and weight loss was assessed over periods <6 months. Finally, the assessment of muscle mass was completed using a subjective physical examination since an objective measure of muscle mass was not available. Nevertheless, physical examination in the form of the PG‐SGA or Subjective Global Assessment (SGA) is commonly used and recommended in clinical practice and therefore the results are clinically relevant.^24^

This study has shown the most important GLIM combinations, when excluding muscle mass assessment, are weight loss and reduced food intake or inflammation (for predicting 30‐day mortality) and weight loss and reduced food intake (for predicting unplanned hospital admission). The accuracy, sensitivity and specificity in predicting adverse outcomes of the machine learning models was unchanged whether all 21 GLIM combinations or the subset excluding muscle mass were used, albeit sensitivity in all models was low. This indicates in healthcare settings where muscle mass is unable to be assessed, its exclusion is unlikely to affect prediction of patients at high risk, supporting the robustness of the GLIM approach and flexibility in excluding certain phenotypic components if needed. However, in this cohort, the exclusion of muscle mass compromised the ability to identify all cases of malnutrition. Future research should focus on consideration of additional clinical variables and whether this improves the sensitivity and ability of the models to determine the direction of the association and examine if these findings are similar in cancer survivors.

## Conflict of interest statement

Dr. Kiss reports grants from Medical Nutrition Industry, grants from Medical Research Future Fund, grants from AuSPEN, grants from Amgen OA‐ANZBMS, grants from Victorian Cancer Agency, outside the submitted work. Irene Deftereos reports grants from AuSPEN and Nestle Health Science, outside the submitted work. Dr. Alizadehsani, Ms. Steer, Ms. Loeliger, Dr. de van der Schueren, Dr. Edbrooke, Dr. Laing and Dr. Khosravi have nothing to disclose.

## Supporting information


**Data S1.** Supporting Information
