# Supplementary material for: Machine learning models to predict outcomes at 30‐days using Global Leadership Initiative on Malnutrition combinations with and without muscle mass in people with cancer
Source: J Cachexia Sarcopenia Muscle. 2023 May 31;14(4):1815–23. doi: 10.1002/jcsm.13259 (PMC10401541; doi:10.1002/jcsm.13259)
Supplement: Supplementary file 1 — Data S1. Supporting Information [file JCSM-14-1815-s001.docx]

**Supplementary Material 1**

| **Combinations with 1 phenotypic and 1 etiologic criteria** | |
| --- | --- |
| GLIM 1 | Weight loss + reduced food intake |
| GLIM 2 | Weight loss + inflammation |
| GLIM 3 | Low BMI + reduced food intake |
| GLIM 4 | Low BMI + inflammation |
| GLIM 5 | Reduced muscle mass + reduced food intake |
| GLIM 6 | Reduced muscle mass + inflammation |
| **Combinations with 1 phenotypic and 2 etiologic criteria** | |
| GLIM 7 | Weight loss + reduced food intake + inflammation |
| GLIM 8 | Low BMI + reduced food intake + inflammation |
| GLIM 9 | Reduced muscle mass + reduced food intake + inflammation |
| **Combinations with 2 phenotypic and 1 etiologic criteria** | |
| GLIM 10 | Weight loss + low BMI + reduced food intake |
| GLIM 11 | Weight loss + low BMI + inflammation |
| GLIM 12 | Weight loss + reduced muscle mass + inflammation |
| GLIM 13 | Weight loss + reduced muscle mass + reduced food intake |
| GLIM 14 | Low BMI + reduced muscle mass + reduced food intake |
| GLIM 15 | Low BMI + reduced muscle mass + inflammation |
| **Combinations with 2 phenotypic and 2 etiologic criteria** | |
| GLIM 16 | Weight loss + reduced BMI + reduced food intake + inflammation |
| GLIM 17 | Weight loss + reduced muscle mass + reduced food intake + inflammation |
| GLIM 18 | Low BMI + reduced muscle mass + reduced food intake + inflammation |
| **Combinations with 3 phenotypic and 1 etiologic criteria** | |
| GLIM 19 | Weight loss + low BMI + reduced muscle mass + reduced food intake |
| GLIM 20 | Weight loss + low BMI + reduced muscle mass + inflammation |
| **Combinations with 3 phenotypic and 2 etiologic criteria** | |
| GLIM 21 | Weight loss + low BMI + reduced muscle mass + reduced food intake + inflammation |
